# Complicated infective endocarditis of the bioprosthetic mitral valve following the transcatheter mitral valve-in-valve procedure: a case report and literature review

**DOI:** 10.1093/ehjcr/ytaf013

**Published:** 2025-01-21

**Authors:** Mohammad Sahebjam, Yeganeh Karimi, Flora Fallah

**Affiliations:** Tehran Heart Center, Cardiovascular Diseases Research Institute, Tehran University of Medical Sciences, North Kargar Ave, Tehran 1411713138, Iran

**Keywords:** Valve-in-valve (ViV) procedure, Prosthetic valve endocarditis, Cardiac abscess

## Abstract

**Background:**

Since the transcatheter valve-in-valve (ViV) procedure was introduced in 2007, a few cases of infective endocarditis (IE) following the ViV procedure have been reported, which can be predisposed by older age, pre-existing medical conditions, and procedural techniques. Paravalvular abscesses constitute a rare complication of IE, resulting from extending IE beyond the valve annulus, less commonly caused by *Klebsiella* species. This complication is more common in prosthetic valves, particularly bioprosthetic valves.

**Case summary:**

We describe a 75-year-old woman with Churg–Strauss syndrome and diabetes mellitus who underwent surgical replacement of bioprosthetic aortic and mitral valves 11 years ago. One year ago, she had a transcatheter mitral ViV procedure due to bioprosthetic mitral valve degeneration. The patient was referred to our centre with fatigue and fever, alongside elevated white blood cell count, erythrocyte sedimentation rate, and C-reactive protein. Blood and urine cultures tested positive for *Klebsiella oxytoca*. Echocardiographic assessments revealed a paravalvular abscess (13 × 8 mm) in the posterolateral side of the bioprosthetic mitral valve, fistulized into the left ventricle. The patient received treatment with vancomycin, meropenem, and colistin and was a candidate for surgery. Eleven days after the patient’s admission, she passed away.

**Discussion:**

This study underscores the novelty of IE complicated with paravalvular abscess following the ViV procedure. In such cases, a multidisciplinary approach and timely surgical interventions are crucial for optimal patient outcomes.

**Learning points**

- As the valve-in-valve (ViV) procedure has been implanted recently for the first time, its related complications and risk factors are less known. Still, infective endocarditis should be considered, particularly in the first year after the procedure.
- Clinicians should be aware of early diagnosis and aggressive treatment, including surgical interventions for managing post-ViV infective endocarditis due to its high mortality rate.

## Introduction

Infective endocarditis (IE) is a severe microbial infection affecting the endocardial surface of both native and prosthetic heart valves. Infective endocarditis can lead to severe complications and has a high mortality rate.^1^ The Global Burden of Disease Study 2019 reported an increase in the incidence of endocarditis, from 478 000 in 1990 to 1 090 530 in 2019.^2^ Approximately 20% of IE cases in 2019 involved prosthetic valve endocarditis.^3^ Individuals with bioprosthetic valves have a higher susceptibility to IE and valve degeneration than those with mechanical alternatives.^4^ Advanced age, pre-existing medical conditions like diabetes mellitus and chronic kidney disease, and immunosuppressed conditions increase the risk of IE.^5,6^ Churg–Strauss syndrome can present with cardiac manifestations, and treatment with immunosuppressive agents can lead to a higher risk of IE.^5,7^

The transcatheter valve-in-valve (ViV) procedure is a minimally invasive treatment option for degenerated bioprosthetic valves.^8^ Although ViV procedures offer advantages in terms of safety and lower mortality rates compared to surgical procedures,^9,10^ paravalvular leaks, transvalvular regurgitation, and higher reintervention rates were observed in such cases.^11^ Nonetheless, the risk of IE following ViV procedures remains unclear due to the novelty of the procedure. Given the higher susceptibility of prosthetic valves to IE,^3^ the presence of more prosthetic surfaces may increase the risk of IE,^12^ evidenced by the higher number of IE cases following the aortic ViV procedure.^13^ Additionally, complex valve anatomy and procedural factors can enhance the IE risk.

A paravalvular abscess is caused by an extension of IE beyond the valve annulus and is more common in prosthetic valves, particularly bioprosthetic valves, due to infection originating at the annulus rather than the leaflet.^14^ Previous studies have reported that 14.4% of patients with IE experience complications due to intracardiac abscesses, which predominantly affect the aortic valve owing to anatomical factors.^15^

The present report describes a patient with Churg–Strauss syndrome and bioprosthetic aortic and mitral valves who developed IE complicated by a paravalvular abscess one year after undergoing a transcatheter ViV procedure.

## Summary figure

**Figure:**
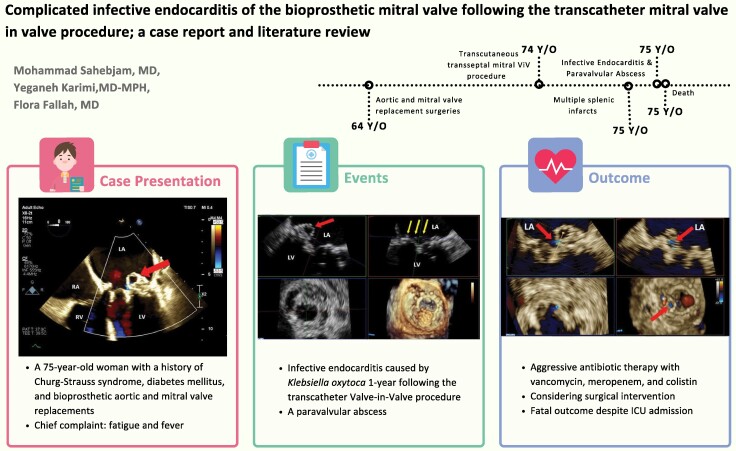


## Case presentation

A 75-year-old woman was referred to our centre with symptoms of fatigue and fever. Her medical history included Churg–Strauss syndrome, managed with 5 mg of prednisolone three times daily, and diabetes mellitus. A month prior, she had been hospitalized with fever and acute abdominal pain, indicating multiple splenic infarcts, which was confirmed by abdominal ultrasonography. Her surgical history included aortic and mitral valve replacement surgeries performed 11 years previously, utilizing PERIMOUNT valves sized 21 and 27, respectively. Due to bioprosthetic mitral valve degeneration (high trans prosthetic gradient and moderate-to-severe transvalvular mitral regurgitation in previous echocardiography), she underwent a transcatheter transseptal mitral ViV procedure using a SAPIEN 3 valve size 26 a year before presentation. Upon admission, the patient presented with a high fever of 40°C, a blood pressure reading of 110/70 mmHg, a heart rate of 113 beats per minute, a respiratory rate of 15 per minute, and a SpO_2_ of 96%. The patient reported exertional dyspnoea classified as functional class II. A cardiac examination revealed a holosystolic murmur at the right lower sternal border and a mid-diastolic murmur at the apex. An ECG demonstrated an atrial fibrillation rhythm. Peripheral and systemic examinations revealed no abnormalities.

Initial laboratory tests showed hyperleukocytosis, with a white blood cell count of 11 900 per µL, anaemia with a haemoglobin level of 8.6 g/dL, a normal platelet count of 123 000 per µL, an elevated erythrocyte sedimentation rate of 114 mm/h, and a moderately elevated C-reactive protein level of 5 mg/L. Additional laboratory data are shown in *Table 1*. Notably, blood cultures tested positive for *Klebsiella oxytoca* three times, as did the urine culture.

**Table 1:** Laboratory and echocardiographic findings

	Actual value	Normal reference range
Laboratory data		
ESR (mm/h)	114	0–22
CRP (mg/dL)	5	<0.5
Urea (mg/dL)	23	16.6–48.5
Cr (mg/dL)	0.8	Female: 0.5–0.9
WBC count (/µL)	11 900	4000–10 000
Hb (g/dL)	8.6	Female: 11–16
Plt count (/µL)	123 000	150 000–450 000
AST (IU/L)	26	Female: up to 32
ALT (IU/L)	27	Female: up to 33
LDH (IU/L)	358	Female: 135–214
Wright test	Negative	
Coomb’s Wright test	Negative	
2ME test	Negative	
Widal test	Negative	
First blood culture	Positive, *Klebsiella oxytoca*	
Second blood culture	Positive, *Klebsiella oxytoca*	
Third blood culture	Positive, *Klebsiella oxytoca*	
Urine culture (mid-stream) (colony count)	Positive, *Klebsiella oxytoca* > 100 000	
Echocardiographic findings		
Left atrial diameter (mm)	55	
Left atrial volume index (mL/m^2^)	81	
Left ventricular diastolic diameter (mm)	50	
Left ventricular systolic diameter (mm)	37	
LVEF (%)	35%	
Mitral valve peak gradient (mmHg)	23	
Mitral valve mean gradient (mmHg)	12	
Mitral valve PHT (ms)	160	
VTI prMV/VTI LVO	3	
Aortic root diameter (mm)	30	
Aortic valve peak gradient (mmHg)	31	
Aortic valve mean gradient (mmHg)	15	
Aortic valve AT (ms)	92	
VTI pr AV/VTI LVO	0.43	
Right atrium volume index (mL/m^2^)	31	
RV S′ wave (cm/s)	6	
TAPSE (mm)	11	
Tricuspid regurgitation gradient (mmHg)	65	
Pulmonary artery pressure (mmHg)	80	
Tricuspid valve annulus (mm)	40	

ESR, erythrocyte sedimentation test; CRP, C-reactive protein; Cr, creatinine; WBC, white blood cell; Hb, haemoglobin; Plt, platelet; AST, aspartate aminotransferase; ALT, alanine transferase; LDH, lactate dehydrogenase; 2ME, 2-mercaptoethanol; LVEF, left ventricular ejection fraction; Mitral valve PHT, mitral valve pressure half-time; VTI pr MV/VTI LVO, velocity time integral prosthetic mitral valve/velocity time integral left ventricle outflow; Aortic valve AT, aortic valve acceleration time; VTI pr AV/VTI LVO, velocity time integral prosthetic aortic valve/velocity time integral left ventricle outflow; RV S′ wave, tissue Doppler peak systolic velocity at the tricuspid annulus; TAPSE, tricuspid annular plane systolic excursion.

**Figure 1:**
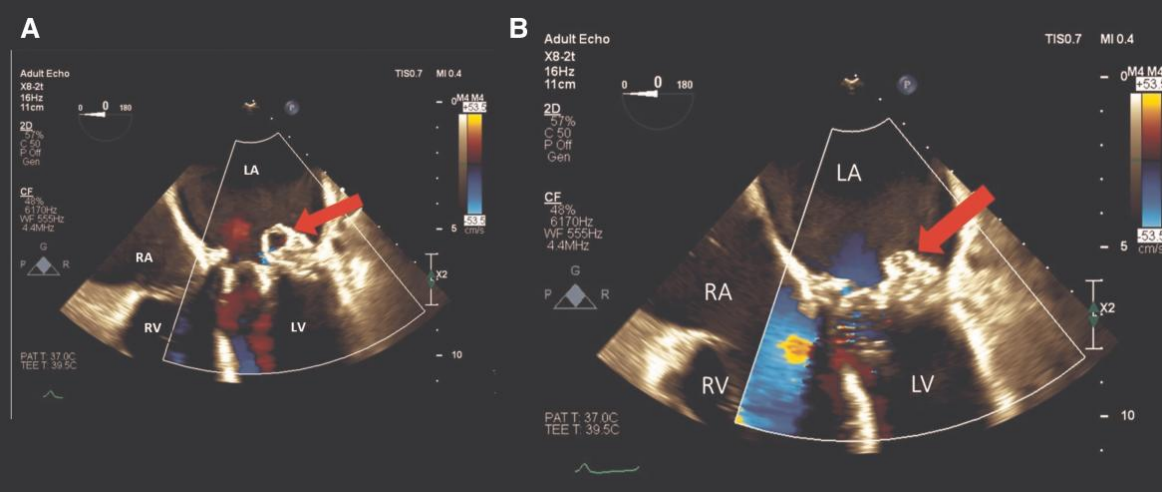
Two-dimensional transoesophageal echocardiography (midoesophageal four-chamber view) with colour flow Doppler demonstrates the bioprosthetic mitral valve abscess expansion in systole (*A*, arrow) and collapse in diastole (*B*, arrow). LA, left atrium; LV, left ventricle; RA, right atrium; RV, right ventricle.

**Figure 2:**
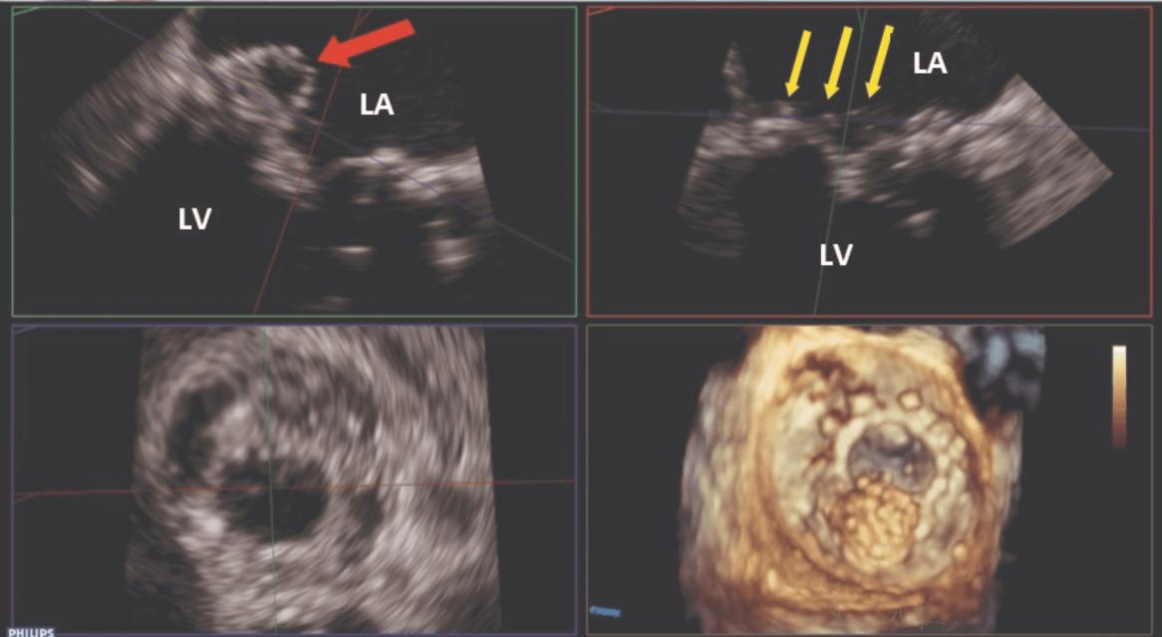
Multiplanar reconstruction of the bioprosthetic mitral valve using transoesophageal echocardiography demonstrates an abscess (red/single arrow) and multiple vegetations (yellow/three arrows). LA, left atrium; LV, left ventricle.

**Figure 3:**
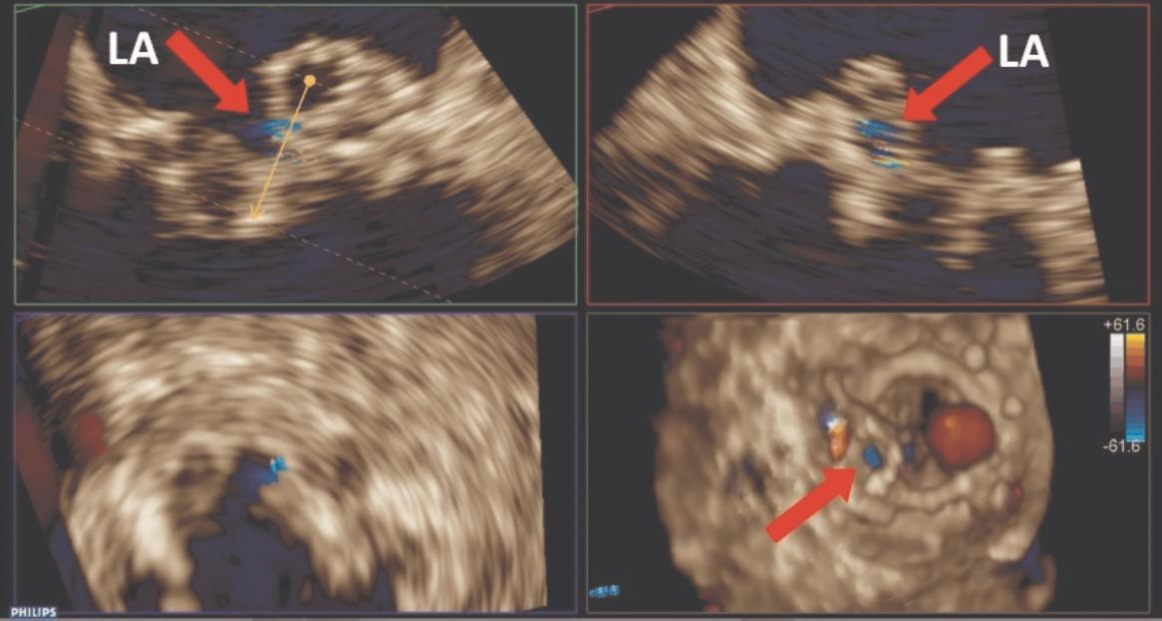
Multiplanar reconstruction of the bioprosthetic mitral valve using transoesophageal echocardiography demonstrates an abscess fistulizing to the left ventricle through an orifice (arrow). LA, left atrium.

The patient underwent transthoracic echocardiography (TTE) and transoesophageal echocardiography (TEE) to investigate the possibility of IE. Transthoracic echocardiography revealed normal left ventricular (LV) size with reduced systolic function (LV ejection fraction = 35%), enlarged right ventricle with impaired systolic function, and enlargement of both atria. Moderate-to-severe tricuspid regurgitation and pulmonary hypertension (pulmonary artery systolic pressure = 80 mmHg) were also observed. The bioprosthetic aortic valve showed normal function, while the transcatheter bioprosthetic mitral valve exhibited significant stenosis and mild transvalvular regurgitation (peak gradient = 23 mmHg, mean gradient = 12 mmHg, and pressure half-time = 160 ms) (*Table 1* and Supplementary material online, *Video S1*). Transoesophageal echocardiography did not reveal paravalvular regurgitation but showed numerous small, hypermobile masses on the atrial side of the bioprosthetic mitral valve, suggesting vegetation. Importantly, a large, pulsatile, hypoechoic cavity (13 × 8 mm) was observed in the posterolateral region of the bioprosthetic mitral valve, indicating a possible valve abscess. Colour flow imaging demonstrated systolic blood flow into the cavity and diastolic flow out of the cavity, denoting a fistulous connection between the abscess and the LV cavity (*Figure 1* and Supplementary material online, *Video S2*). A mobile mass was also detected on the atrial surface of the cavity, likely representing vegetation. Additionally, the TEE demonstrated mild thickening of the bioprosthetic aortic valve leaflets with mild transvalvular regurgitation and no visible mass. For a more comprehensive evaluation of the bioprosthetic mitral valve, 3D echocardiography was performed, revealing the abscess cavity, vegetations, and fistulous connection to the LV cavity (*Figures 2* and *3* and Supplementary material online, *Videos S3–S6*). According to the Duke criteria for IE, the presence of one major (evidence of endocardial involvement by echocardiography) and four minor criteria (predisposing heart condition, fever, vascular phenomenon, positive blood culture but not meeting major criteria) confirmed the diagnosis of mitral valve IE with no involvement of other valves. Based on the clinical presentation and paraclinical findings, the treatment was started on intravenous antimicrobial therapy at admission, consisting of vancomycin 1 g twice daily and cefepime 1.5 g three times daily. After 2 days, cefepime was replaced with meropenem 2 g three times daily and colistin 4.5 MIU twice daily (plus a 9 MIU loading dose) to address mitral bioprosthetic IE complicated by a paravalvular abscess. The regimen of vancomycin, meropenem, and colistin was maintained until the patient’s final day of hospitalization. Although the patient was a candidate for cardiac surgery, her family declined the procedure. Due to respiratory distress, the patient required intubation and mechanical ventilation and was subsequently admitted to the ICU. Unfortunately, she experienced cardiac arrest and expired a day later.

## Discussion

This case presents a rare occurrence of a paravalvular abscess one year after the transcatheter ViV procedure in a patient with a bioprosthetic mitral valve infected by *K. oxytoca*. The ViV procedure, first implemented in 2007,^16^ has not been clearly established, so few complications, particularly post-procedure IE, have been reported.^11,17–19^ From five documented IE cases after the transcatheter mitral ViV, one case presented with early IE two months after the ViV procedure,^17^ while others presented after eight months^20^ and one^11^ and two years.^18^ Moreover, one of them was suffering from systemic lupus erythematosus and was under rheumatological medications,^18^ and the other one had mild to moderate intraventricular mitral regurgitation after the ViV procedure.^20^ Therefore, the first year of post-procedure,^11,18^ immunocompromised condition,^18^ and valvular regurgitation^20,21^ might contribute to IE development. In terms of complications, one patient presented IE and a pseudoaneurysm at the apex of the left ventricle,^17^ one complicated with stroke,^20^ and another complicated with severe mitral stenosis required redo surgery.^18^ No paravalvular abscess has been reported yet. Notably, the complicated cases with pseudoaneurysm at the apex of the left ventricle and stroke underwent transapical ViV procedure.^17,20^ Although transapical access offers better control over valve implantation, patients with this access are at higher risk of one-year mortality than patients with transseptal access.^22^ Still, there is no evidence to compare IE risk between these two access sites. However, there is not enough supporting evidence for the priority of transseptal access over transapical, as some studies reported no differences in mortality.^11,23^ Transseptal transcatheter aortic valve implantation showed a higher risk of post-procedural complications, like stroke.^24^ Regardless of access route in ViV procedures, several studies showed reduced complications, including stroke, bleeding, acute kidney injury, arrhythmias, permanent pacemaker insertion, and left ventricular outflow tract obstruction, compared to redo surgery.^9,10,25^

Paravalvular abscesses, albeit infrequent, are life-threatening.^15^ Common causative agents for bioprosthetic valve infections include alpha-haemolytic streptococci, enterococci, and coagulase-negative staphylococci.^19^  *Klebsiella* species, such as *K. oxytoca*, are less frequently associated with such infections. A recent systematic review identified 67 cases of IE caused by *Klebsiella* species, including 11 cases involving prosthetic valves. Only 13 cases were complicated by paravalvular abscesses, with *Klebsiella pneumoniae* being the primary causative agent.^26^ Contrasting to our patient infected by *K. oxytoca*, other IE cases post-transcatheter mitral ViV were infected by *Staphylococcus aureus*,^11,17^ coagulase-negative staphylococci,^11^ and *Candida albicans*.^18^

Risk factors for IE and paravalvular abscesses post-ViV include patient-related factors such as diabetes and immunocompromised states (e.g. Churg–Strauss syndrome),^26^ as well as procedural techniques.^14^ Preventive strategies like antibiotic prophylaxis, postoperative monitoring for signs of infection, and timely interventions are crucial in preventing abscess development.^27^

Diagnosing paravalvular abscesses is challenging due to their rarity and non-specific clinical manifestations. Nevertheless, echocardiographic techniques, particularly TEE and 3D echocardiography, play a crucial role. Transoesophageal echocardiography offers high sensitivity (76%–100%) and specificity (95%) in diagnosing IE on bioprosthetic valves, which is further enhanced by 3D echocardiography.^28^ The combined use of TEE, spectral, and colour Doppler methods also aids in identifying fistulas and pseudoaneurysms.^14^ Due to the high mortality associated with paravalvular abscesses, timely administration of broad-spectrum antibiotic therapy for a minimum of 6 weeks is essential.^29^ Surgical consultations are advisable at the time of diagnosis, as delayed interventions may increase morbidity and mortality.^30^ Surgical interventions should be considered for most patients, except those with abscesses smaller than 1 cm and without complications such as heart block, valvular dehiscence, or insufficiency.^14^ While a few cases of survival without surgical intervention have been reported,^29^ the operative mortality rate remains high for active IE with paravalvular abscesses, leading to poor short-term and long-term prognoses.^30^

This report aligns with a documented case by Vendramin *et al.*,^19^ where an 83-year-old man with a history of bioprosthetic aortic valve replacement later underwent a ViV procedure with a CoreValve prosthesis due to bioprosthesis degeneration. After 4 years, the patient presented with IE complicated by a periannular abscess and vegetation on the bioprosthetic aortic valve. The patient reoperation and broad-spectrum antibiotic therapy and survived. Mubarak^18^ reported a case of IE 2 years after a transcatheter mitral ViV procedure. Their patient was a 47-year-old woman with a history of systemic lupus erythematosus. She had undergone bioprosthetic mitral valve replacement 9 years before the study. Two years before the study, she had also received a transcatheter ViV implantation. She underwent a successful 6-month course of antifungal therapy in addition to receiving a mitral valve replacement. Additionally, a 72-year-old man presented IE and pseudoaneurysm at the apex of the left ventricle three months after the mitral ViV procedure.^17^ The patient presented with IE, exhibiting fever, purulent discharge at the site of thoracotomy, and 15 mm mobile vegetation on the leaflet of the prosthetic valve, as observed in both transthoracic echocardiogram (TTE) and transoesophageal echocardiogram (TEE). Positive blood cultures confirmed *S. aureus*. The patient was treated with co-trimoxazole for 6 weeks, clindamycin for 1 week, and underwent pseudoaneurysm closure. The patient was discharged after 15 days, asymptomatic.

## Conclusions

This case underscores the challenges in managing IE in patients with bioprosthetic valves, particularly following the transcatheter ViV procedure. It highlights the critical role of early diagnosis and aggressive treatment in such cases. As the ViV procedure is a novel technique with limited documented cases, post-ViV IE is not well-understood. Given the poor outcomes and high mortality rate associated with these cases, a multidisciplinary approach, along with surgical interventions when appropriate, should be considered for optimal management.

## Lead author biography


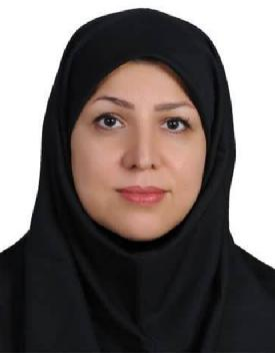


Dr Flora Fallah is an echocardiologist and currently works at the Tehran Heart Center Hospital. She completed her cardiac specialty at Shahid Beheshti University in 2009 and later graduated with an echocardiology fellowship from Tehran University of Medical Sciences in 2014.

## Supplementary Material

ytaf013_Supplementary_Data

## Data Availability

Data supporting the findings of this study are available from the corresponding author upon reasonable request.

## Notes

**Consent:** The authors confirm that the patient has provided written consent for the submission and publication of this case report, including images and associated text, in adherence to COPE guidelines.

## Notes

**Funding:** The authors received no financial support for the research, authorship, and/or publication of this article.
